# Therapeutic CK2 inhibition attenuates diverse prosurvival signaling cascades and decreases cell viability in human breast cancer cells

**DOI:** 10.18632/oncotarget.2248

**Published:** 2014-07-23

**Authors:** G. Kenneth Gray, Braden C. McFarland, Amber L. Rowse, Sara A. Gibson, Etty N. Benveniste

**Affiliations:** ^1^ Department of Cell, Developmental and Integrative Biology, University of Alabama at Birmingham, Birmingham, Alabama, USA

**Keywords:** breast cancer, CK2, STAT3, NF-κB

## Abstract

Breast cancer is the most common malignancy in women worldwide and remains a major cause of mortality, thus necessitating further therapeutic advancements. In breast cancer, numerous cell signaling pathways are aberrantly activated to produce the myriad phenotypes associated with malignancy; such pathways include the PI3K/Akt/mTOR, NF-κB and JAK/STAT cascades. These pathways are highly interconnected, but one prominent lateral enhancer of each is the remarkably promiscuous kinase CK2. CK2 expression has been shown to be elevated in cancer, thus implicating it in tumorigenesis through its effects on oncogenic signaling cascades. In this study, we identify aberrant expression of CK2 subunits in human breast samples from The Cancer Genome Atlas dataset. Additionally, two specific small molecule inhibitors of CK2, CX-4945 and TBB, were used to examine the role of CK2 in two human breast cancer cell lines, MDA-MB-231 and MCF-7 cells. We show that CK2 inhibition attenuates constitutive PI3K/Akt/mTOR, NF-κB and STAT3 activation and inducible NF-κB and JAK/STAT activation and downstream transcriptional activity. CX-4945 treatment caused a range of phenotypic changes in these cell lines, including decreased viability, cell cycle arrest, apoptosis and loss of migratory capacity. Overall, these results demonstrate the tremendous potential of CK2 as a clinical target in breast cancer.

## INTRODUCTION

Breast cancer is the most commonly diagnosed cancer in women worldwide and remains a clinically challenging disease because of distant metastases and the extreme heterogeneity of tumors referred to as “breast cancer.” Recent classification studies have proposed 10 and 4 molecular subtypes of breast cancer [1, 2], and neither classification scheme fully accounts for the complexity of the diverse cellular signaling pathways driving cancer growth and progression. Numerous pathways, such as the NF-κB, JAK/STAT and PI3K/Akt pathways, help drive at least some breast tumors. This extreme diversity points to the need to continue developing molecularly targeted therapies.

JAK/STAT signaling is intimately involved in both normal mammary development and mammary tumor formation [3]. Of the seven STATs, STAT3 is by far the most prominent in cancer [4]. STAT3 upregulates a broad repertoire of genes with diverse functions, including cell growth (c-Myc), apoptosis resistance (Bcl-2), migration/invasion (MMP2, 9), angiogenesis (VEGF) [5] and inflammation (IL-6) [4]. Activated STAT3 thus has a striking ability to promote cancer cell survival, invasion, and stemness while suppressing anti-tumor immunity [4, 6]. In accordance with STAT3's pro-cancerous properties, constitutive tyrosine phosphorylation of STAT3 is observed in nearly a third of breast adenocarcinomas and is an indicator of poor prognosis [7]. STAT3 activation is also associated more with advanced breast cancer, as opposed to STAT5, which is more prominently involved in tumor initiation [3].

NF-κB is a family of transcription factors composed of five members, the most important of which in cancer is the p65 (RelA) and p50 heterodimer. This dimer is normally found in the cytoplasm, as it is sequestered there by the Inhibitor of NF-κB (IκB) proteins. Activation of the pathway by a stimulus such as TNF-α causes activation of the IKK complex, which phosphorylates IκB, thus targeting it for ubiquitination and proteasomal degradation. Then, p65 is phosphorylated at serine 536 and activated; it can then translocate into the nucleus, causing transcription of numerous genes such as IL-6 and IL-8 [8]. A myriad of NF-κB target genes have been implicated in cancer pathogenesis and progression. Indeed, NF-κB itself has been demonstrated numerous times to drive pro-cancerous inflammation, prosurvival signals and radiation resistance [9, 10].

The PI3K/Akt/mTOR pathway is one of the most commonly dysregulated pathways in cancer. This prosurvival signaling cascade can be activated in response to a variety of stimuli, including cytokines and growth factors [11]. Genetic lesions in genes associated with this pathway, such as *PIK3CA* and *PTEN*, are among the most common such aberrations in breast cancer [2]. As such, inhibition of this pathway is a clinical priority, particularly as overactivation of the PI3K/Akt/mTOR pathway has been associated with drug resistance in breast cancer [12, 13].

CK2 is a constitutively active, ubiquitous serine/threonine kinase most often found as a tetramer composed of two catalytic subunits (α and/or α’) and two regulatory subunits (the β subunit). CK2 is highly pleiotropic [14]: it has nearly 450 known substrates [15] and may target over 20% of the phosphoproteome, according to a recent bioinformatic analysis [16]. CK2 is also found in nearly every subcellular compartment [17], and knockout of CK2α or CK2β is embryonic lethal [18, 19]. Given the enormous importance of CK2 in normal cells, its importance in neoplastic cells may hardly be surprising. CK2 levels are elevated in every cancer type evaluated to date [14], including breast cancer [20]. CK2 seems to be a superb example [14] of non-oncogene addiction [21], as it enhances every one of Hanahan and Weinberg's hallmarks of cancer [22-24] and underpins multi-drug resistance [14, 25]. CK2 is a “lateral” (non-hierarchical and therefore atypical) kinase [26] that affects numerous pathways vital in cancer [22].

We recently discovered that CK2 is a novel interaction partner with JAK1 and JAK2. CK2 inhibition was thus able to prevent STAT3 activation and downstream gene activation in various cell types, including triple-negative breast cancer cells [27]. CK2 is also known to target the NF-κB pathway at multiple points, including IκBs, IKKs and p65 itself [28]. The PI3K/Akt/mTOR pathway is also intimately intertwined with CK2. CK2 phosphorylates Akt at serine 129, causing Akt to become hyperactive [22]; Akt also phosphorylates CK2, causing CK2 to promote rRNA synthesis [29]. PTEN is a CK2 target, and its modulation causes stabilization and (somewhat paradoxically) inactivation of PTEN [30].

Cylene Pharmaceuticals developed a first-in-class, orally bioavailable inhibitor of CK2 [31]. This highly selective drug, called CX-4945 (Silmitasertib) is the first CK2 inhibitor to enter clinical trials [32]. *In vitro* studies of CX-4945 provide evidence for its ability to attenuate diverse pro-cancerous signaling pathways and to decrease breast cancer cell viability in a manner positively correlating with the CK2 levels of the specific cell line [31]. CX-4945 also decreases IL-6 serum levels and STAT3 levels in an inflammatory breast cancer model [33]. We have demonstrated that CX-4945 decreases NF-κB, PI3K/Akt and JAK/STAT3 signaling in glioma and increases survival time in an intracranial murine model of glioma [34]. Finally, recent data from a phase I clinical trial in solid tumors initiated by Cylene Pharmaceuticals show that CX-4945 treatment, which produced minimal side-effects, reduced circulating tumor cell count and CK2-related pro-cancerous signaling while also stabilizing disease in a fifth of patients in a way that strongly correlated with decreased IL-6 and IL-8 levels [35]. These extremely promising results point to the extreme importance of both CK2 and its numerous interwoven signaling targets in tumor growth and progression.

In this study, we identify widespread genetic aberrations in CK2 genes in human breast cancers in a subtype-specific manner and characterize CK2 protein levels in two human breast cancer cell lines. We demonstrate that small molecule inhibition of CK2 by CX-4945 and TBB can attenuate an array of constitutive signaling pathways as well as inducible JAK/STAT and NF-κB signaling. Finally, we show that inhibition of CK2 with CX-4945 causes cell cycle arrest and decreased cell viability in human breast cancer cell lines, as well as changing cell morphology and migratory capacity. CK2 thus appears to be a vital foundation of multiple aspects of cancer cell biology and a target worthy of further investigation.

## RESULTS

### CK2 Subunits Are Differentially Upregulated in Human Breast Cancers

**Figure 1:**
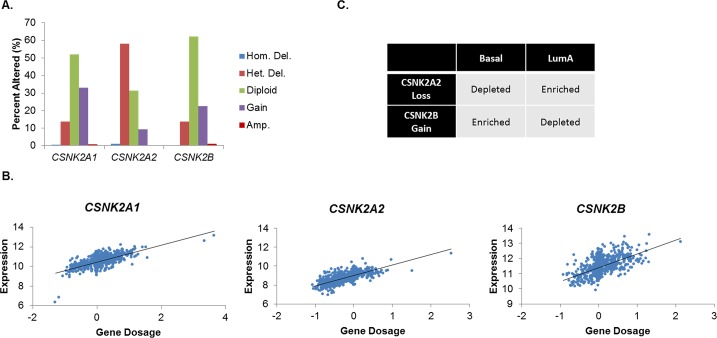
CK2 Subunit Expression Is Differentially Elevated in Human Breast Cancers A, Analysis of *CSNK2A1*, *CSNK2A2*, and *CSNK2B* copy number in the Breast TCGA dataset (n=1,061). Copy number was determined by cBioPortal using the GISTIC algorithm. B, Gene dosages of *CSNK2A1*, *CSNK2A2*, and *CSNK2B* were plotted against mRNA expression (z-score) of each respective gene in order to determine the significance of copy number to gene expression (p<10^−20^ in all cases). C, Depletion/enrichment of *CSNK2A2* loss and *CSNK2B* gain in Basal and Luminal A breast cancers. Depletion/enrichment was determined by chi-squared analysis (n=485; all p<10^4^).

The statuses of the CK2 subunits were initially analyzed in human breast cancer from The Cancer Genome Atlas (TCGA) [2]. A large fraction of tumors demonstrate copy number variation (CNV) in one or more CK2 genes (Figure 1A). Approximately 30% and 20% of breast tumors have gains on *CSNK2A1* (encoding CK2α) and *CSNK2B* (CK2β), respectively, while fewer gains are seen on *CSNK2A2* (CK2α’). Unexpectedly, a large number of tumors also possess heterozygous deletions of CK2 genes: most prominently, *CSNK2A2* is lost in nearly 60% of tumors. Losses at *CSNK2A1* and *CSNK2B* are more modest (~15%). The correlation between copy number and mRNA expression was also examined, and it was found that copy number significantly correlated with expression for all three genes (p<10^−20^), as shown in Figure 1B.

In order to better understand this unusual distribution of CNV, CNV was examined by breast cancer molecular subtype (Luminal A, Luminal B, Her2-enriched and Basal). Figure 1C shows significant depletions/enrichments exist for two subtypes, Luminal A and Basal. Luminal A appears to be enriched for *CSNK2A2* loss and depleted for *CSNK2B* gains, whereas Basal is exactly reversed. No significant enrichments/depletions were observed for *CSNK2A1*. These results would suggest that Basal breast tumors would possess higher levels of CK2 as compared to non-Basal, and indeed, higher expression of all three subunits is observed in this subtype (data not shown). These data are intriguing because they suggest that the Basal subtype, which has fewer treatment options and therefore a more dire prognosis [2], may be highly susceptible to CK2 inhibition.

### Characterization of CK2 Status in Human Breast Cancer Cell Lines

**Figure 2:**
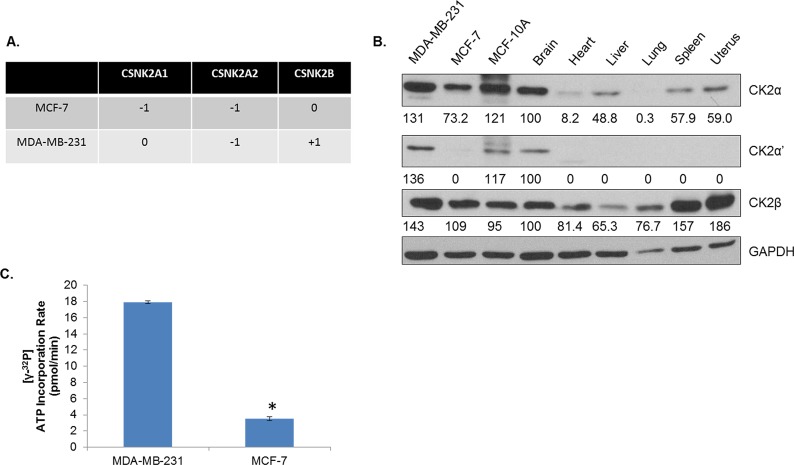
CK2 Expression Is Elevated in Human Breast Cancer Cells Compared to Normal Tissue A, Copy number variations of *CSNK2A1*, *CSNK2A2*, and *CSNK2B* were determined by interrogating the Cancer Cell Line Encyclopedia by cBioPortal using the GISTIC algorithm. B, Levels of CK2α, α’ and β were examined by immunoblot for MDA-MB-231 and MCF-7 human breast cancer cells, MCF-10A immortalized human breast epithelium cells, and perfused, normal murine tissues, including brain, heart, liver, lung, spleen and uterus. Densitometry was performed, and normal brain was used to determine fold change. C, CK2α and CK2α’ were pulled down from 200 μg of lysate from MDA-MB-231 and MCF-7 cells. The immunoprecipate was then used in a CK2 activity assay which measures incorporation of ^32^P from [γ-^32^P]ATP into a CK2 substrate peptide using a scintillation counter. Assay performed in duplicate. *, p<.001.

In order to evaluate the therapeutic potential of CK2 inhibition, we utilized two commonly used human breast cancer cell lines, MDA-MB-231 and MCF-7 cells. The former is triple negative, while the latter is hormone receptor positive. Considering the interesting contrasts in CK2 subunit expression described in Figure 1, we sought to profile CK2 status in these two breast cancer lines. First, CNV for MCF-7 and MDA-MB-231 was examined for CK2 genes using the Cancer Cell Line Encyclopedia (Figure 2A) [36]. Both cell lines demonstrate heterozygous loss of *CSNK2A2*. MCF-7 cells additionally have a heterozygous loss of *CSNK2A1*, while MDA-MB-231 cells have gains on *CSNK2B*. Next, in Figure 2B, CK2α, α’ and β expression was compared for MDA-MB-231, MCF-7, MCF-10A (an immortalized breast epithelium line) and various normal murine tissues. It has been previously reported that CK2 expression/activity is highest in the brain [37], and our results confirm that CK2 expression in the brain is high. Indeed, among normal tissue, CK2α’ was only detectable in the brain. However, the three altered breast lines seem to have extraphysiological expression of CK2, even in comparison with the brain, thus confirming that CK2 expression is elevated in cancerous tissues.

A CK2 kinase assay was performed in order to determine the catalytic activity of CK2 in the MDA-MB-231 and MCF-7 cell lines. CK2α and CK2α’ were immunoprecipitated from protein lysates (200 μg) from each cell line, and as seen in Figure 2C, MDA-MB-231 cells have a far higher (>4 times) rate of CK2 kinase activity than MCF-7 cells.

### CK2 Inhibition Causes Widespread Attenuation of Constitutive Prosurvival Signaling

**Figure 3:**
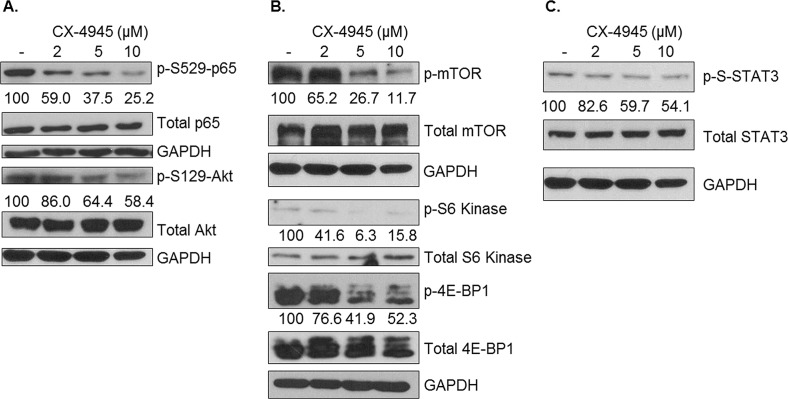
Pharmacological CK2 Inhibition Broadly Attenuates Constitutive Cell Signaling MDA-MB-231 cells were treated for 4 h in serum-free medium with CX-4945 at 2, 5 and 10 μM. Lysates were obtained, and immunoblots performed using the indicated antibodies to examine effects on NF-κB and Akt (A), mTOR (B) and STAT3 (C) signaling components. GAPDH was used as a loading control for each protein located above it in the panels. Densitometric quantifications for each phosphoprotein are shown, with untreated cells serving as the reference point (100%); percent inhibition is shown. Experiments were repeated with similar results, and representative data are shown.

After having characterized the relative levels of the various CK2 subunits and CK2 enzymatic activity in human breast cancer cell lines, we sought to establish the cell signaling consequences of CK2 inhibition on the line possessing the highest CK2 expression and activity, MDA-MB-231. Multiple signaling pathways are known to drive breast cancer, including MAPK, Akt/mTOR, NF-κB and JAK/STAT. In order to evaluate these signaling pathways' response to CK2 inhibition, MDA-MB-231 cells were treated with 2, 5 and 10 μM of CX-4945 for 4 h, and components of each of the above pathways were evaluated by immunoblotting. As shown in Figure 3, CX-4945 inhibition of CK2 resulted in widespread decreases in activation of each these pathways. To confirm that CK2 was indeed inhibited by CX-4945 treatment, p-S529-p65 and p-S129-Akt levels were evaluated (Figure 3A), as these phosphorylation sites are known CK2-specific sites. Serine 529 phosphorylation is associated with full transcriptional capacity of p65, and serine 129 phosphorylation of Akt is thought to produce a hyperactive state in Akt [22]. Constitutive phosphorylation of both of these sites was dose-dependently decreased by CX-4945 treatment (Figure 3A). Activation of molecules downstream of Akt was also evaluated (Figure 3B). For example, mTOR phosphorylation decreased in response to CX-4945, and targets of mTORC1 - S6 kinase and 4E-BP1 - were also shown to have decreased phosphorylation upon CK2 inhibition.

In Figure 3C, it is shown that constitutive p-S-STAT3 is inhibited at higher concentrations of CX-4945 in MDA-MB-231 cells. Although the tyrosine phosphorylation of STAT3 is usually associated with STAT3 activation and oncogenic activity, MDA-MB-231 cells have very low levels of constitutive p-Y-STAT3; however, p-S-STAT3 is considered necessary for full transcriptional activation of STAT3 [38, 39], as well as its oncogenic mitochondrial activity [40]. Phosphorylation at serine 727 on STAT3 was shown to be MEK1,2-dependent; accordingly, activation of the MEK1,2 target ERK1,2 was evaluated and was inhibited by CX-4945 treatment (data not shown). In general, Figure 3 shows a broad attenuation of constitutive cell signaling in human breast cancer cells upon CK2 inhibition.

### Loss of CK2 Activity Prevents Full Activation of STAT3 and Target Gene Transcription

**Figure 4:**
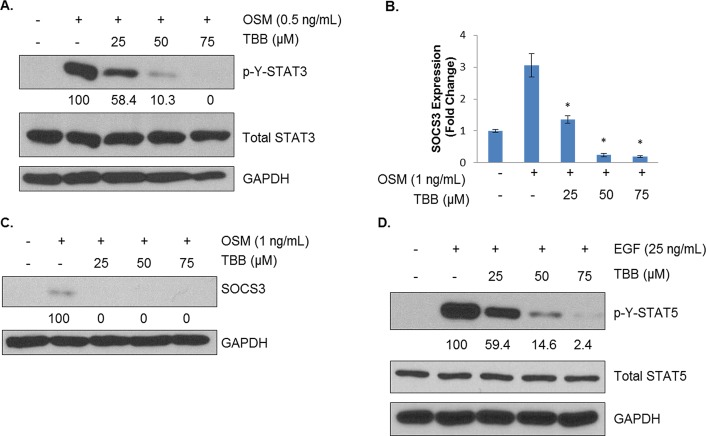
CK2 Is Necessary for Full Activation of Cytokine- and Growth Factor-Induced STATs A, MDA-MB-231 cells were treated for 2 h in serum-free medium with TBB at 25, 50 and 75 μM and then treated for 15 minutes with 0.5 ng/mL of OSM. Lysates were obtained, and immunoblots performed using the indicated antibodies. B, MDA-MB-231 cells were treated for 2 h in serum-free medium with TBB at 25, 50 and 75 μM and then treated for 1 h with 1 ng/mL of OSM. RNA was isolated, cDNA was generated, and q-RTPCR was performed for *SOCS3*. PCR was performed in triplicate: *, p<0.001. C, MDA-MB-231 cells were treated for 2 h in serum-free medium with TBB at 25, 50 and 75 μM and then treated for 1 h with 1 ng/mL of OSM. Lysates were obtained, and immunoblots performed using the indicated antibodies. D, MDA-MB-231 cells were treated for 2 h in serum-free medium with TBB at 25, 50 and 75 μM and then treated for 30 minutes with 25 ng/mL of EGF. Lysates were obtained, and immunoblots performed using the indicated antibodies. Densitometric quantifications for each phosphoprotein are shown, with cells treated with cytokine alone serving as the reference point (100%); percent inhibition is shown. All experiments were repeated, and representative data are shown.

Although examination of constitutive cell signaling is important for determining CK2's control of the phosphoproteome, *in vivo* cancer cells exist in a supportive stroma bathed in a highly complex milieu of cytokines, chemokines and growth factors. Therefore, the effects of CK2 inhibition on inducible cell signaling were examined. Previously, we had shown that CK2 is necessary for full activation of STATs in hematological malignancy [27] and glioblastoma [34], and we sought to examine this in breast cancer. As shown in Figure 4A, OSM potently induces tyrosine phosphorylation of STAT3 in MDA-MB-231 cells. However, pretreatment of these cells with TBB, another CK2 small molecule inhibitor [22], strongly blocks STAT3 activation in a dose-dependent fashion. In order to determine the functional relevance of this effect, expression of SOCS3, an endogenous negative regulator of STAT3 transcriptionally controlled by STAT3, was determined. As shown in Figure 4B, *SOCS3* levels were induced threefold in response to OSM treatment, and TBB treatment impressively decreased *SOCS3* transcript levels such that at 50 and 75 μM, *SOCS3* transcription is below basal levels. Consistent with this observation, SOCS3 protein levels were strongly inhibited by CK2 inhibition (Figure 4C).

We also sought to determine whether the CK2-dependency of STAT activation held for other activators of STAT. MDA-MB-231 cells were treated with EGF for 30 minutes, with or without 2 h of TBB pretreatment. As shown in Figure 4D, EGF caused very strong activation of STAT5, another oncogenic STAT family member [3]. TBB pretreatment blocked full activation of STAT5 in a dose-dependent manner. Similar results were obtained for STAT3 activation by LIF and IL-6 trans-signaling (data not shown). These results indicate that CK2's effects on STAT activation are broad-ranging and affect multiple STAT proteins using distinct receptors for activation.

### TNF-α-induced NF-κB Activation and Target Gene Transcription Relies on CK2

**Figure 5:**
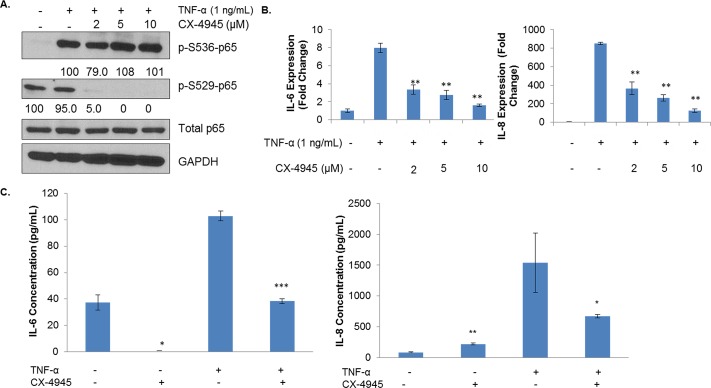
CK2 Inhibition Affects the Phosphorylation State and Transcriptional Capacity of TNF-α-induced NF-κB A, MDA-MB-231 cells were treated for 4 h in serum-free medium with CX-4945 at 2, 5 and 10 μM and then treated for 15 minutes with 1 ng/mL of TNF-α. Lysates were obtained, and immunoblots performed using the indicated antibodies. Densitometric quantifications for each phosphoprotein are shown, with cells treated with cytokine alone serving as the reference point (100%); percent inhibition is shown. B, MDA-MB-231 cells were treated for 4 h in serum-free medium with CX-4945 at 2, 5 and 10 μM and then treated for 1 h with 1 ng/mL of TNF-α. RNA was isolated, cDNA was generated, and q-RTPCR was performed for the indicated genes. PCR was performed in triplicate: **, p<0.001. C, MDA-MB-231 cells were treated for 48 h with 1 ng/mL of TNF-α and/or 10 μM of CX-4945. Supernatants were collected, and ELISA was performed for the indicated cytokines. ELISA performed in triplicate: *, p≤0.01; **, p<0.001; ***, p<0.0001. All experiments were repeated, and representative data are shown.

Next, we evaluated the potential of CK2 to affect TNF-α-induced NF-κB signaling. As previously mentioned, serine 529 phosphorylation, which is controlled by CK2, is associated with full transcriptional activity of p65, but serine 536 phosphorylation is the canonical marker of NF-κB activation in response to external factors and stressors, such as TNF-α. Although CK2 does not directly phosphorylate serine 536, its actions on IκBs and IKKs may provide an indirect mode of regulation [28]. MDA-MB-231 cells were pretreated with CX-4945 at varying concentrations for 4 h, after which they were treated with 1 ng/mL of TNF-α for 15 minutes. In contrast with some previous reports in other cell lines [34, 41], serine 529 phosphorylation was not affected by cytokine stimulation, although it was strongly inhibited by CX-4945 (Figure 5A). Serine 536 was strongly induced by TNF-α treatment, but CX-4945 evoked little attenuation of this site's levels.

CK2 inhibition seems to have an interesting, conflicting effect on the post-translational modification landscape of p65, and so we sought to determine what, if any, effect this may have on the transcription of NF-κB target genes. We treated MDA-MB-231 cells with CX-4945 at varying concentrations for 4 h, followed by 1 h of TNF-α stimulation. We then examined *IL-6* and *IL-8* transcript levels, two NF-κB target genes whose circulating blood levels have been correlated with clinical response to CX-4945 [33, 35]. Somewhat surprisingly, CK2 inhibition dramatically decreased transcription of these genes. Even at low levels of CX-4945, *IL-6* and *IL-8* transcription decreased by over 50% (Figure 5B). Because of these strong effects at the transcriptional level, IL-6 and IL-8 protein levels were examined by ELISA. MDA-MB-231 cells were treated for 48 h without or with TNF-α and/or CX-4945. Supernatants were collected, and secreted cytokine quantified. As shown in Figure 5C, CX-4945 blocked production of these two interleukins in response to TNF-α by approximately 50%.

### CK2 Inhibition Results in Cell Cycle Arrest and Loss of Viability

**Figure 6:**
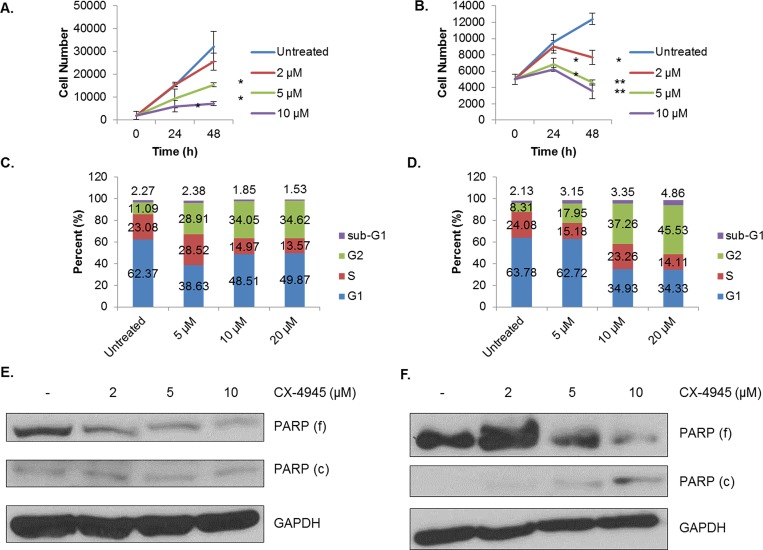
CX-4945 Causes Cell Cycle Arrest and Loss of Viability A-B, MDA-MB-231 (A) and MCF-7 (B) cells were plated at 2,000 and 5,000 cells per well, respectively, and treated with CX-4945 at 2, 5 and 10 μM. The WST-1 assay was performed at 0, 24 and 48 h. The assay was performed in triplicate at each time and concentration: *, p<0.05; **, p<0.005. C-D, MDA-MB-231 (C) and MCF-7 (D) cells were treated for 24 h with CX-4945 at 5, 10 and 20 μM. Cells were then fixed, stained with propidium iodide (PI), and analyzed by flow cytometry in order to determine cell cycle stage. E-F, MDA-MB-231 (E) and MCF-7 (F) cells were treated for 24 h with CX-4945 at 2, 5 and 10 μM. Lysates were obtained, and immunoblots performed using the indicated antibodies. All experiments were repeated, and representative data are shown.

As the above data demonstrate, CK2 has multiple effects on the signaling landscape of human breast cancer cell lines. We were therefore interested in the functional consequences of CK2 inhibition on these cell lines *in vitro*. First, we performed the WST-1 cell viability assay using CX-4945 dose- and time-dependently. As shown in Figure 6A, MDA-MB-231 cells nearly sextupled over the course of 72 h, while MCF-7 cells grew much more slowly, not even doubling in the same period (Figure 6B). Despite these strong differences in growth rate, CK2 inhibition seemed to strongly inhibit growth, starting at 24 h and persisting through 72 h. Indeed, the more aggressive MDA-MB-231 line was more sensitive to CX-4945, with absorbance at 72 h being barely within the detectable range.

An important issue with the WST-1 assay, however, is that it only indirectly measures cell growth. It directly measures metabolic activity, and CK2, in its prodigious promiscuity, has multiple documented effects on cell metabolism [26, 42]. We therefore sought to broaden the measures by which we evaluated CK2 inhibition's effect on cancer cells. We performed cell cycle analysis on both the MDA-MB-231 and MCF-7 lines using propidium iodide staining. Figure 6C-D show that for both cell lines, 24 h of CX-4945 causes a dose-dependent increase in the proportion of cells in G2, with a concurrent decrease in the percentage of cells in G1. This suggests that CK2 inhibition causes a G2/M arrest, which is in line with our previous reports in glioblastoma [34].

However, in order to determine whether loss of CK2 activity causes actual cell death, we treated both cell lines with CX-4945 and evaluated PARP status by immunoblotting. As shown in Figures 6E-F, at 24 h, CX-4945 causes dose-dependent decreases in full-length PARP, although increases in cleaved PARP were only observed in the MCF-7 cell line. This finding suggests these human breast cancer cells undergo cell death in response to CK2 inhibition, as well as cell cycle arrest.

### CK2 Inhibition Affects Breast Cancer Cell Morphology and Migratory Capacity

**Figure 7:**
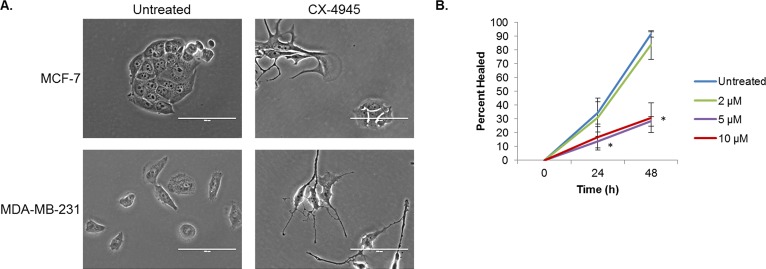
CX-4945 Affects Cell Morphology and Wound Healing A, MCF-7 and MDA-MB-231 cells were treated with 10 μM of CX-4945 for 18 h and photographed at 40X. Scale bar is 100 μm. B, MDA-MB-231 cells were plated at confluency and allowed to adhere. Cells were then scratched once horizontally and thrice vertically with a p200 pipette tip. They were allowed to heal for 48 h in 1% serum-containing medium with CX-4945 at 2, 5 and 10 μM. Pictures of each cross were taken at 0, 24 and 48 h, and the percent healed area was analyzed by TScratch software. As indicated, each condition was done in triplicate: *, p<0.002. Each experiment was repeated, and data shown are representative.

In addition to its effects on viability, we observed that treatment with CX-4945 has striking effects on the morphology of both the MDA-MB-231 and MCF-7 cell lines, which is in keeping with multiple reports of CK2's vital role in maintenance of the cytoskeleton [43]. Figure 7A compares control cells from both lines with those which have been treated for 18 h with 10 μM of CX-4945. Both cell lines exhibit signs of cytoplasmic retraction and cell rounding in response to CK2 inhibition.

Finally, considering its major effects on cell morphology, we were interested in the potential of CK2 to affect cell migration. To this end, a wound healing assay was performed on the MDA-MB-231 cells (Figure 7B), which were plated at confluency, scratched with a p200 pipette tip, and allowed to heal for 48 h in 1% serum-containing medium. At 24 h, higher concentrations of CX-4945 (5 and 10 μM) were able to significantly inhibit wound healing, and this difference became much more striking at 48 h. These results indicate not only that is CK2 necessary for cell cycle progression, viability and morphological maintenance but also that it may also support the invasive potential of breast cancer cells.

## DISCUSSION

In this paper, we highlight the pervasive dependency of malignant breast cancer cells on CK2 activity for constitutive signaling, cell cycle progression and cell survival. First, we unveiled widespread aberrations in CK2 gene dosages. While gains in CK2α and CK2β were common, the most common aberration was heterozygous loss of CK2α’. This result is somewhat puzzling, as CK2α’ possesses many of the same catalytic properties as the highly homologous CK2α subunit, while also having distinct pro-tumorigenic targets and effects [44]. Thus, determining the pathophysiological role of *CSNK2A2* loss in breast cancer is important, as it may provide further information on the distinct roles of the two CK2 catalytic subunits. Following the bioinformatic analyses, both the triple negative MDA-MB-231 and HR+ MCF-7 lines were shown to display elevated CK2α and CK2β. The MDA-MB-231 cells, however, have much higher levels of CK2α’ and higher CK2 kinase activity than MCF-7 cells. This finding is interesting principally because it opens the possibility that triple negative tumors, which are extremely difficult to treat in comparison with other clinical subtypes of breast cancer, will be especially sensitive to therapeutic CK2 inhibition.

Having thus described the CK2 status in human breast cancers, we examined the signaling consequences of pharmacological inhibition of CK2. Treatment with CX-4945 caused a broad attenuation of various prosurvival signaling cascades, including STAT3, Akt/mTOR and NF-κB, the clinical targetings of which have had limited success. These results strongly support the role of CK2 as an underlying bolster to cell signaling generally. However, from a therapeutic standpoint, these results are especially exciting, as they suggest that CK2 inhibition may be a means of indirectly inhibiting numerous procancerous survival pathways by means of a single drug, rather than the numerous agents which would be necessary to inhibit each pathway individually.

We further sought to describe CK2's role in inducible STAT signaling. We found that CK2 inhibition prevents full activation of STAT3 in response to several IL-6 family cytokines and STAT5 in response to EGF. Additionally, the transcription and translation of the STAT3 target gene *SOCS3* was attenuated by CK2 inhibition, supporting the functional consequences of STAT3 inhibition. These results have clinical significance, as STAT3 activation has been associated with late-stage, metastatic breast cancer [45, 46]. Thus, CK2 inhibition may be able to functionally replace inhibitors of STAT3, which have had difficulty reaching the clinic [47].

According to our results, CK2 is also necessary for the transcriptional activity of NF-κB in breast cancer, although it is not strictly necessary for the canonical activation of this same transcription factor. These results are intriguing, as CK2 has been previously reported as an enhancer of signaling upstream of NF-κB p65 [28]. However, in MDA-MB-231 cells, CK2 inhibition does not strongly affect serine 536 phosphorylation, the canonical marker of NF-κB activation and transcriptional capacity. Instead, CX-4945 dose-dependently decreases constitutive serine 529 phosphorylation, which in turn correlates with decreases in transcription of the NF-κB target genes *IL6* and *IL8*. These results support the hypothesis that CK2 acts by various noncanonical avenues to affect its target pathways and raises the possibility that CK2 constitutively primes p65 for transcriptional activity in response to various stimuli. This result is intriguing from a therapeutic standpoint, as it suggests that NF-κB inhibition may be achieved through alternative means, a welcome possibility considering the many difficulties in producing a clinically viable NF-κB inhibitor [9]. Indeed, there is some indirect evidence that the clinical response to CX-4945 is connected to NF-κB signaling. Phase I studies have shown that patients have high circulating levels of IL-6 and IL-8 prior to treatment with CX-4945 and that treatment with CX-4945 causes decreases in the circulating levels of these cytokines [35]. This decrease may be important in itself, since both of these cytokines have been connected to late stage disease, stemness and metastasis [6, 48, 49] as well as the tumor self-seeding phenomenon [50]. That high NF-κB activity may be the key factor in determining response to CK2 inhibition is an intriguing hypothesis worthy of further investigation.

Finally, we demonstrate that CX-4945 treatment causes, in both MDA-MB-231 and MCF-7 cells, cell cycle arrest, morphological changes and apoptosis. In addition, MDA-MB-231 cells have a decrease in migratory capacity in response to CK2, potentially implicating this kinase in the metastatic niche. These results collectively support the concept of CK2 as vital for cancer cell survival and aggressiveness.

In conclusion, our results have added to the growing body of literature supporting the potential of CK2 as a target in a wide variety of cancers, particularly breast cancer. CK2's role in supporting numerous signaling cascades and its importance to various functional properties of breast cancer cells highlight it as an important future target.

## MATERIALS AND METHODS

### Cells and Reagents

MDA-MB-231 triple-negative human breast cancer cells were obtained from the ATCC and maintained in DMEM medium supplemented with 10% fetal bovine serum (FBS), 1 mM sodium pyruvate, 2 mM L-glutamine, 100 U penicillin/mL and 100 μg streptomycin/mL. MCF-7 ER/PR+ human breast cancer cells were obtained from the ATCC and maintained in MEM medium supplemented with the above reagents as well as 1X non-essential amino acid mixture (Lonza) and 0.01 mg/mL human recombinant insulin. Recombinant human TNF-α, IL-6, sIL-6R and OSM were purchased from R&D Systems, and recombinant human EGF was purchased from Miltenyi Biotec Inc. Antibodies against phospho-STAT3 Y705, phospho-STAT3 S727, STAT3, phospho-STAT5 Y694, STAT5, phospho-p70 S6 kinase S371, p70 S6 kinase, phospho-4E-BP1 T37/46, 4E-BP1, phospho-mTOR S2448, mTOR, phospho-p65 S536, AKT, phospho-p44/42 MAPK T202/Y204 (p-ERK1,2), and p44/42 (ERK1,2) were purchased from Cell Signaling Technology. Antibodies against p65, SOCS3, phospho-c-Jun, c-Jun, CK2α and CK2α’ were purchased from Santa Cruz Biotechnology. Antibodies against GAPDH and phospho-p65 S529 were purchased from Abcam, and the antibody against CK2β was purchased from Calbiochem. The antibody against phospho-AKT S129 was purchased from Abnova, and the antibody against PARP was purchased from BD Biosciences. CX-4945, a CK2 inhibitor, was synthesized and generously provided by Cylene Pharmaceuticals [31]. TBB was purchased from EMD Biosciences. The Millipore Casein Kinase 2 Assay Kit was used to determine CK2 enzymatic rates.

### Immunoblotting

Cells were serum-starved during treatment and lysed in RIPA buffer with protease and phosphatase inhibitors. Protein concentration was determined using the BioRad Assay. Equivalent amounts of total protein (10-30 μg) were analyzed by SDS-PAGE with antibodies specified above, as previously described [51].

### Total RNA Isolation and Quantitative RT-PCR

Total RNA was isolated as previously described [51]. Complementary DNA was synthesized and analyzed by quantitative PCR as previously described [52] using the following primers/probe sets purchased from Applied Biosystems: *IL-6* (Hs00174131_m1)*, SOCS3* (Hs02330328_s1)*, IL-8* (Hs00174103_m1) and *18S* (Hs99999901_s1).

### ELISA

Cells were serum-starved for 24 h during treatment without or with TNF-α and/or CX-4945. Supernatants were collected, and levels of secreted IL-6 and IL-8 were quantified by ELISA (Biolegend).

### Functional Assays and Flow Cytometry

For cell viability/metabolism, cells were plated in 96 well plates at a density of 2,000 cells/well for MDA-MB-231 cells and 5,000 cells/well for MCF-7 cells. The cells were then treated with the indicated doses of CX-4945 for 48 h, and the WST-1 cell viability assay was performed as previously described [52]. For the wound healing assay, MDA-MB-231 cells were plated at confluency and were then scratched once horizontally and thrice vertically using a p200 pipette tip. They were then rinsed twice with PBS and put in 1% serum DMEM with the indicated concentrations of CX-4945. The cells were imaged at 0, 24 and 48 h, and the unhealed area was quantified using TScratch software, developed by the Koumoutsakos group (CSE Lab) at ETH Zürich [53]. For cell cycle analysis, cells were treated with CX-4945 for 24 h, fixed with 70% methanol for 15 minutes, stained with propidium iodide (PI) while incubating with RNase A and examined by flow cytometry. Data were analyzed using FlowJo (TreeStar), and cell cycle distribution was determined using the Dean-Jett-Fox model.

### Densitometry, Bioinformatics and Statistical Analysis

For densitometry, immunoblots were scanned, and the density of each lane of phosphorylated and total protein was quantified. Phosphorylated protein densities were normalized to their respective total protein densities, and they were then converted to a percent of the appropriate control. For bioinformatic analyses, both Oncomine and cBioPortal were utilized to access TCGA data [54, 55]. For statistical analyses, ANOVA analysis was done on appropriate multivariable analyses with Student-Newman-Keuls post hoc analysis, and students t-test for comparison of 2 conditions. Values represent the mean ± SD unless noted otherwise. p< 0.05 was considered statistically significant unless stated otherwise.
